# Supplementary material for: NRK1 controls nicotinamide mononucleotide and nicotinamide riboside metabolism in mammalian cells
Source: Nat Commun. 2016 Oct 11;7:13103. doi: 10.1038/ncomms13103 (PMC5476803; doi:10.1038/ncomms13103)
Supplement: Supplementary Information — Supplementary Figures 1-9, Supplementary Table 1 and Supplementary Methods [file ncomms13103-s1.pdf]

Supplementary Figure 1

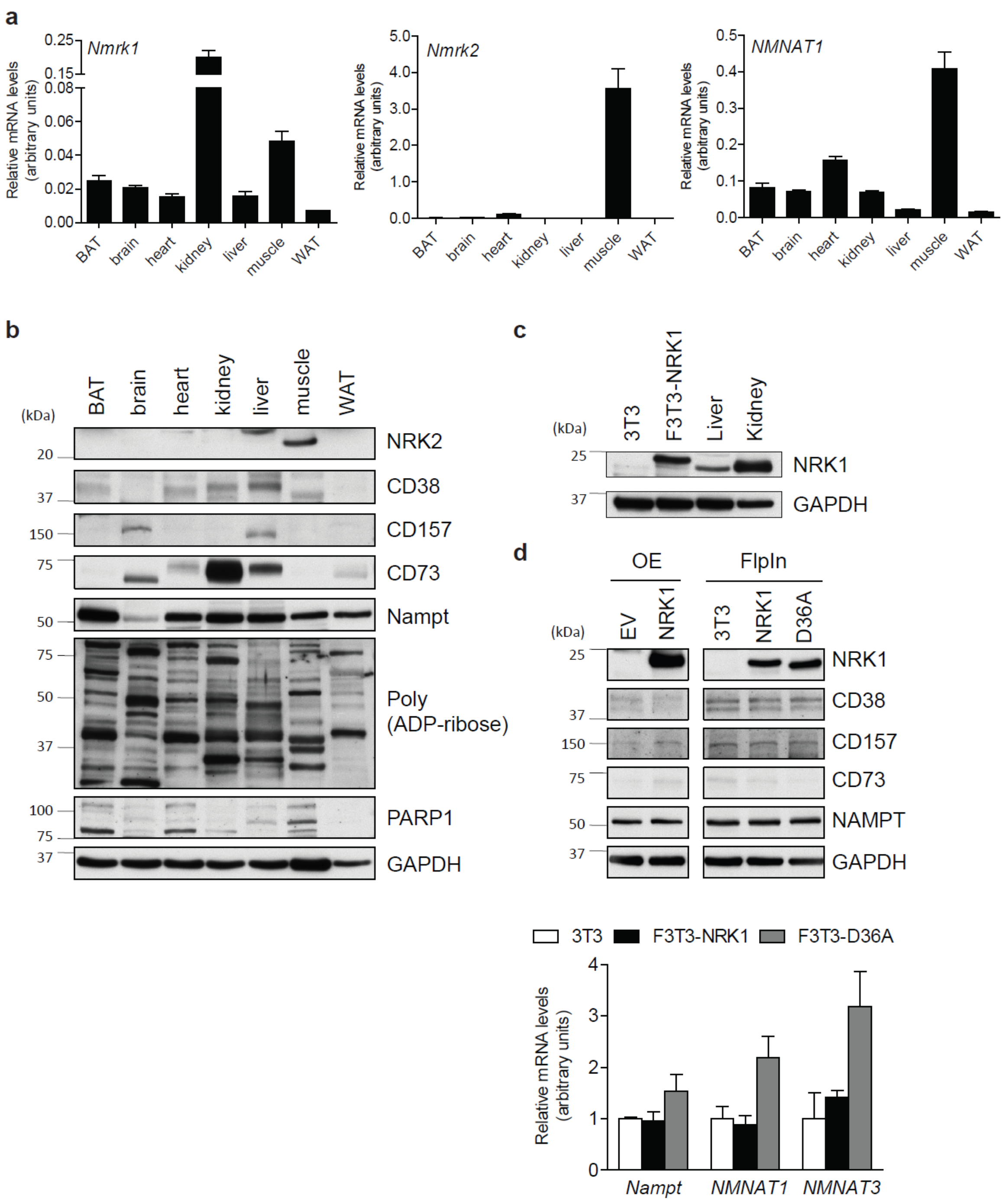

Supplementary Figure 1. NAD$^+$-related genes expression in mouse tissues and in cells overexpressing NRK1. mRNA **(a)** and protein **(b)** levels of NRK1, NRK2 and other enzymes involved in NAD$^+$ metabolism in selected tissues of WT mouse. BAT – brown adipose tissue, WAT – white adipose tissue. **(c)** F3T3-NRK1 cell line expresses NRK1 protein levels comparable to endogenous levels from WT mouse. **(d)** Western blot and mRNA levels of NAD$^+$ metabolism related enzymes in transient overexpression of NRK1 in NIH/3T3 cells (OE) and in F3T3 cells. Results shown are mean ± SEM. (n=3)

Supplementary Figure 2

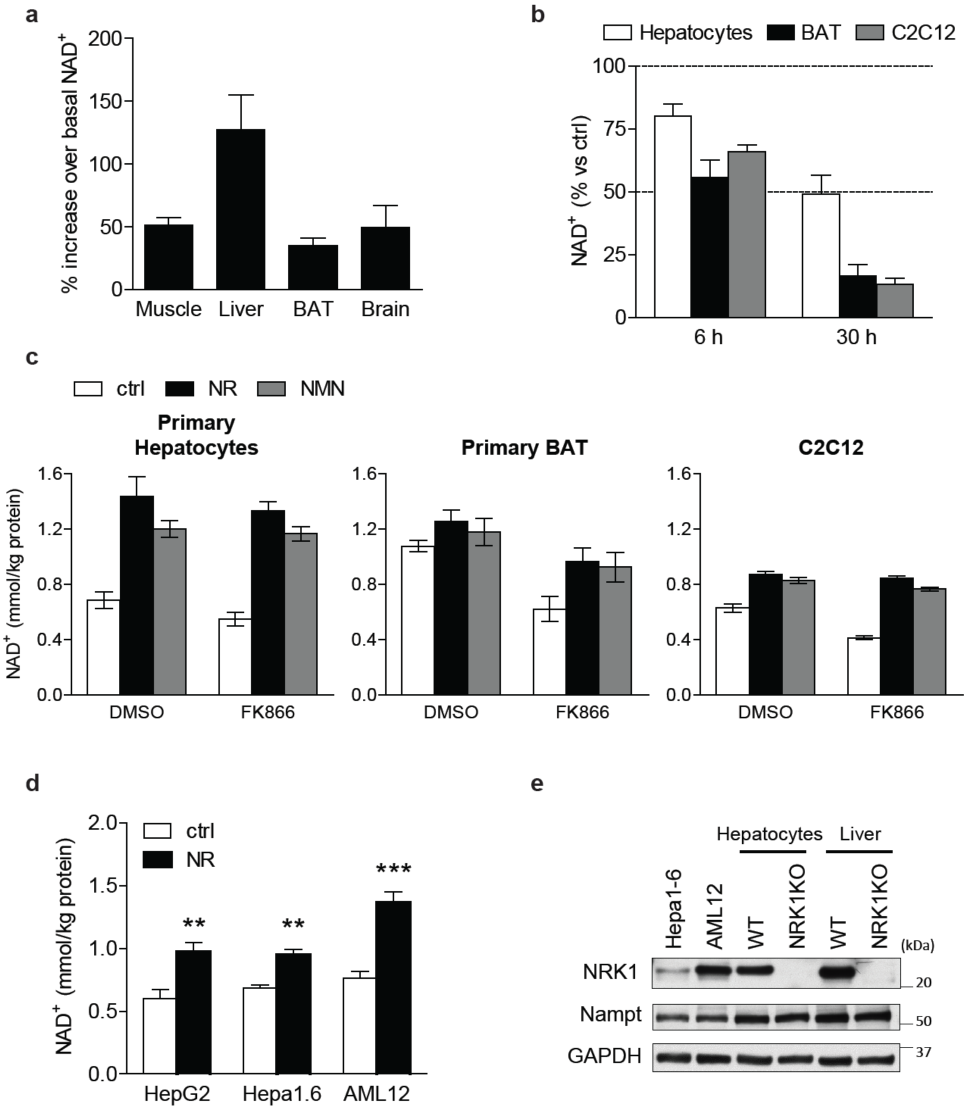

Supplementary Figure 2. NRK1 and NR metabolism in hepatic cells. (a) 5-6 weeks old WT male mice were IP injected with 500 mg/kg of NR or vehicle and tissues were collected after 1 hour. Percentage increase above basal NAD^+ level upon NR treatment in selected tissues, n=5 per group. **(b-c)** Primary hepatocytes and primary brown adipose cells (BAT) isolated from WT mice, as well as C2C12 cells, were treated with 1 hour or 24 hours of 2μM FK866 followed by supplementation with 0.5 mM NR, 0.5 mM NMN or vehicle control for 6 hours. **(b)** Basal NAD^+ levels in different cell types after FK866 treatment. **(c)** NAD^+ increase upon NR and NMN supplementation is not affected by FK866. **(d)** NAD^+ measurement in 6 hours 0.5 mM NR-treated HepG2, Hepa1.6 and AML12 cells. **(e)** NRK1 protein levels in Hepa1.6 and AML12 compared to the levels in liver and primary hepatocytes. Results shown are mean ± SEM, * p<0.05 ** p<0.01 *** p<0.001 vs ctrl. (n=3)

Supplementary Figure 3

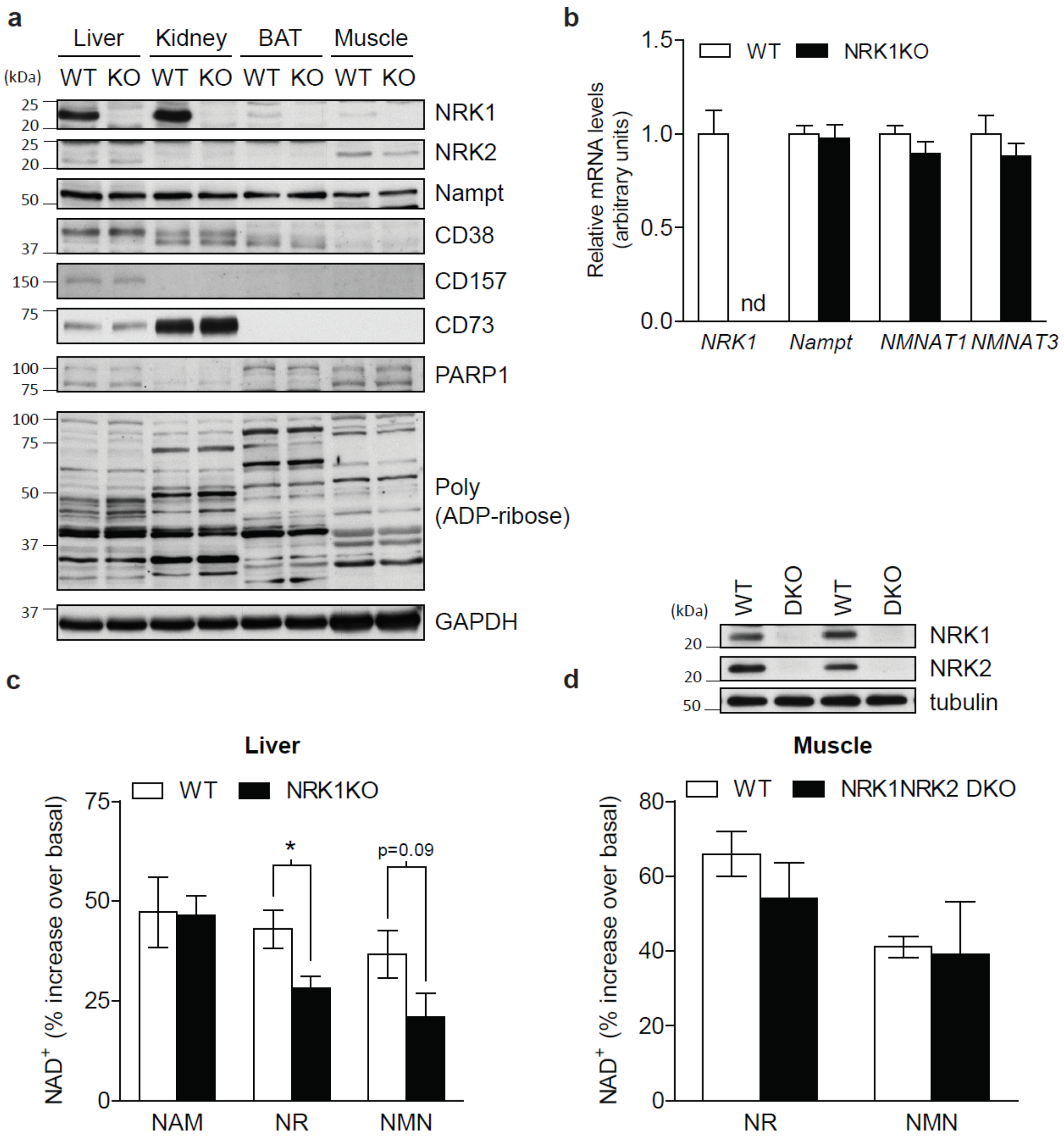

Supplementary Figure 3. NRK1KO mouse validation and NAD$^+$ *in vivo* metabolism in NRK1 defective mice. (a) NRKs and other NAD$^+$-related enzymes protein expression in liver, kidney, brown adipose (BAT) and skeletal muscle from WT and NRK1KO male mice. **(b)** mRNA levels of NAD$^+$-related enzymes in liver of *Nmrk1*-deficient mice**. (c)** 5-6 weeks old WT and NRK1KO male mice were IP injected with 50 mg/kg of the indicated compounds. Then, liver samples were collected after 1 hour, n=5 per group. Percentage increase above basal NAD$^+$ level in liver tissue upon treatments. **(d)** 7-8 weeks old WT and NRK1NRK2 DKO male mice were IP injected with 500 mg/kg of NR or NMN. Then, skeletal muscle samples were collected after 1 hour, n=3 per group. Percentage increase above basal NAD$^+$ level in muscle upon treatments. Results shown are mean ± SEM, * $p<0.05$.

Supplementary Figure 4

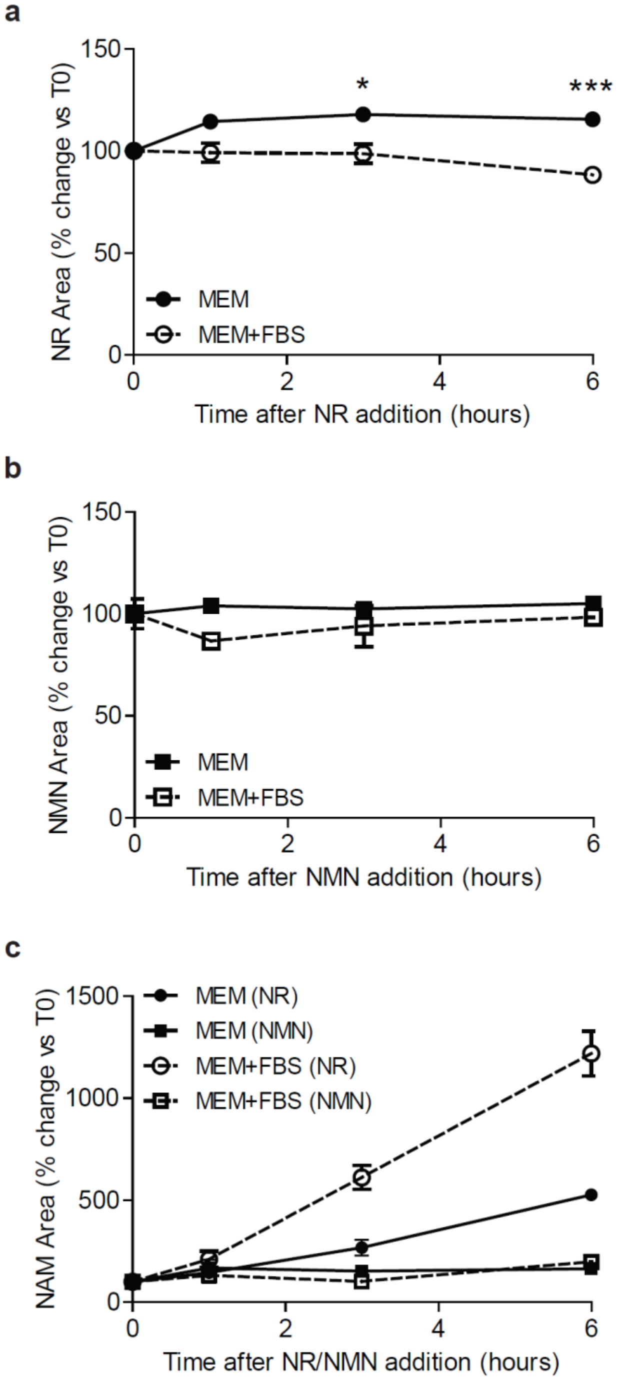

Supplementary Figure 4. NR and NMN stability in cell culture medium. 0.5 mM NR or 0.5 mM NMN was incubated for 6 hours with plain NAM-free Minimal Essential Media (MEM, solid line) or media supplemented with 10% FBS (MEM+FBS, dashed line). Percentage of remaining NR **(a)** and NMN **(b)** in the samples and percentage of accumulative increment of NAM **(c)** versus time point T=0. Results shown are mean ± SEM, * $p<0.05$ *** $p<0.001$ vs WT. (n=3)

Supplementary Figure 5

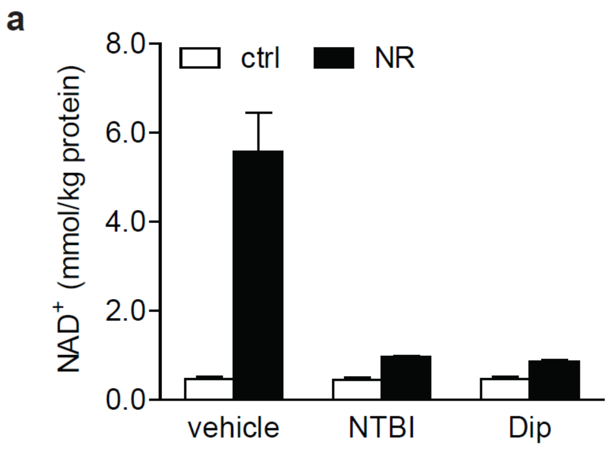

Supplementary Figure 5. ENTs inhibition prevents NR-induced NAD^+ synthesis in F3T3-NRK1 cells. (a) NAD^+ measurement in F3T3-NRK1 cells treated for 1 hour with ENTs inhibitors NTBI (10 µM) or dipyridamole (Dip) (2 µM) followed by 6 hours treatment with 0.5 mM NR. Results shown are mean ± SEM. (n=3)

Supplementary Figure 6

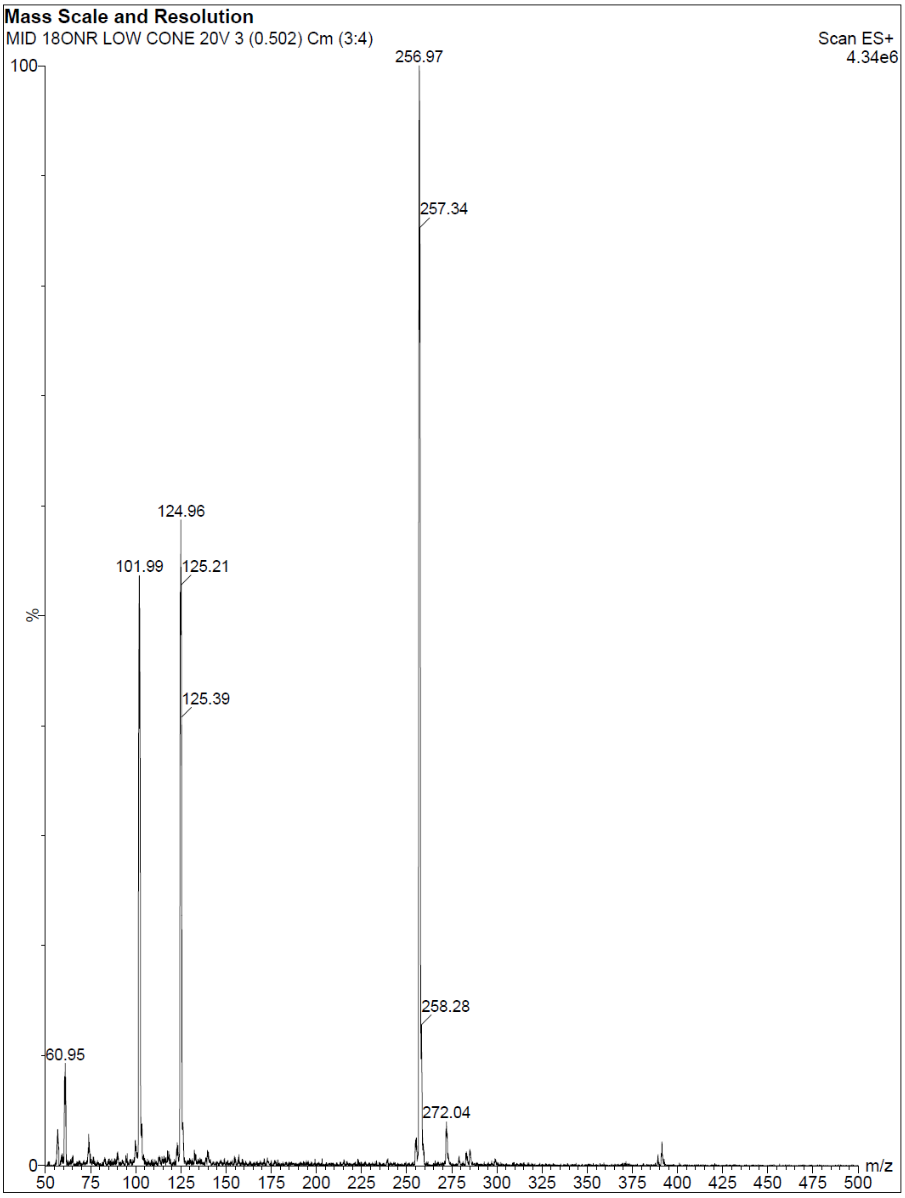

Supplementary Figure 6. Multiple ion detection mass spectrum of ^{18}O-labeled NR. The analyte was infused on a PHD 2000 infusion instrument (Harvard Apparatus) with an infusion rate of 20 µl/min and coupled to a Waters Acquity TQD mass spectrometer (Milford, MA, USA) with an electrospray ionization source operating in positive mode. Instrument settings were as follows: source temperature was set at 150ºC, cone gas flow at 100 $L.h^{-1}$, desolvation temperature at 350ºC and desolvation gas flow at 700 $L.h^{-1}$. The capillary voltage was set at 2.9 kV in positive mode. Mass spectra data were acquired in continuum mode using a cone voltage of 20V over the range m/z 50-500 with a scan time of 10s.

Supplementary Figure 7

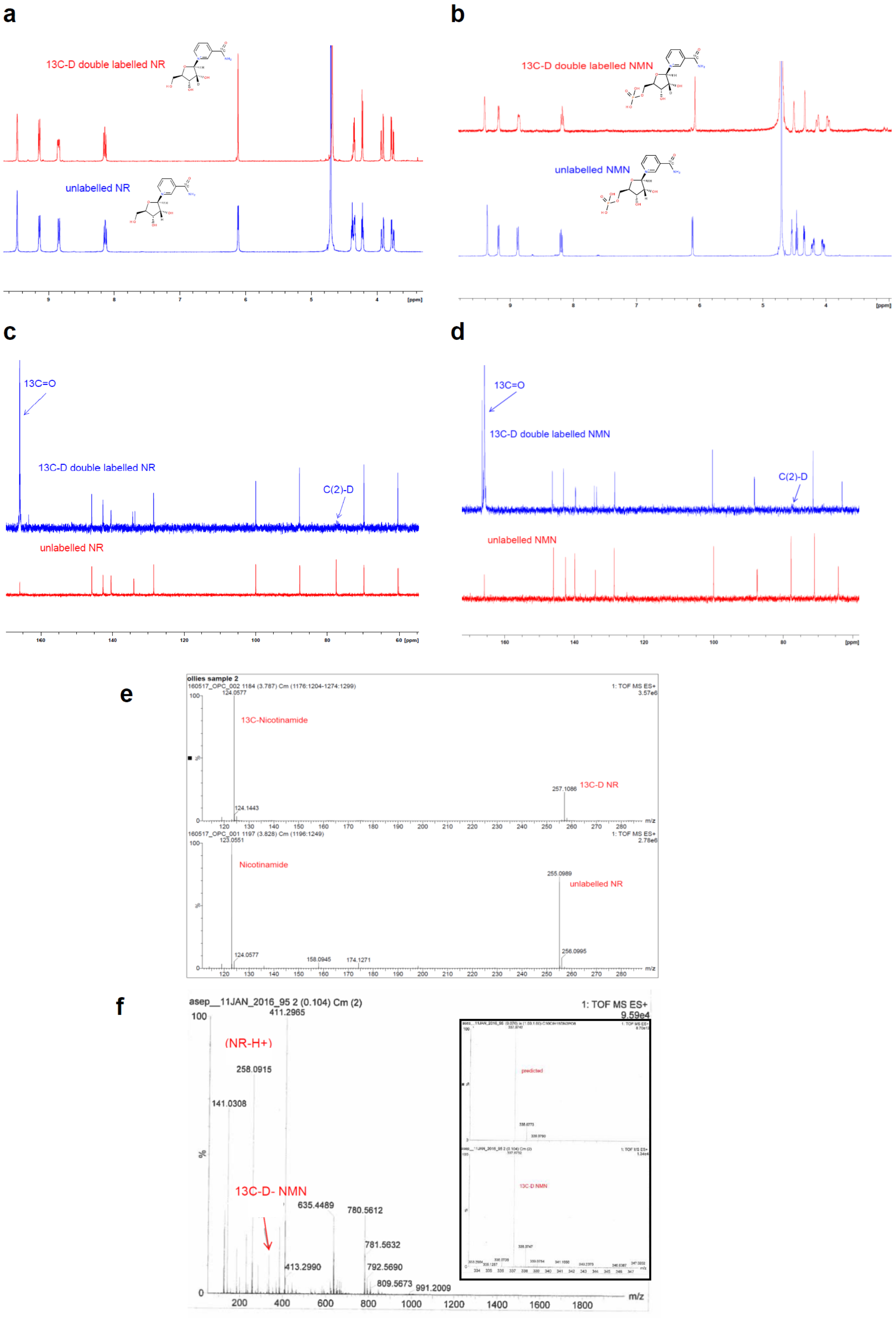

Supplementary Figure 7. Analytical report on double labeled and unlabeled NR and NMN. **(a)** ^{1}H NMR spectrum taken in D_2O of unlabeled NR (blue) and of ^{2}H, ^{13}C labeled NR (red); The exchange of $^{1}H_{2'}$ for $^{2}H_{2'}$ results in the disappearance of the doublet of doublet at 4.19ppm characteristic of $^{1}H_{2'}$ and a change in the splitting pattern of the peaks corresponding to $^{1}H_{1'}$ and $^{1}H_{3'}$ while the incorporation of a ^{13}C in the carbonyl of the nicotinamide moiety results in a new splitting pattern for the signal of H_4 **(b)** ^{1}H NMR spectrum taken in D_2O of unlabeled NMN (blue) and of ^{2}H, ^{13}C labeled NMN (red); The exchange of $^{1}H_{2'}$ for $^{2}H_{2'}$ results in the disappearance of the doublet of doublet at 4.42ppm characteristic of $^{1}H_{2'}$, a change in the splitting pattern of the peaks corresponding to $^{1}H_{1'}$ and $^{1}H_{3'}$ while the incorporation of a ^{13}C in the carbonyl of the nicotinamide moiety results in a new splitting pattern for the signal of H_4. **(c)** ^{13}C NMR spectrum taken in D_2O of unlabeled NR (red) and of ^{2}H, ^{13}C labeled NR (blue); The exchange of $^{1}H_{2'}$ for $^{2}H_{2'}$ results in the appearance of a triplet corresponding to $C_{2'}$ due to the $^{2}H_{2'}$-$C_{2'}$ coupling while the incorporation of a ^{13}C in the carbonyl (C=O) of the nicotinamide moiety results in a ^{13}C-^{13}C splitting pattern for the signal corresponding to C_3 (NR: 134ppm) and C_4 (NR: 141ppm); **(d)** ^{13}C NMR spectrum taken in D_2O of unlabeled NMN (red) and of ^{2}H, ^{13}C labeled NMN (blue); The exchange of $^{1}H_{2'}$ for $^{2}H_{2'}$ results in the appearance of a triplet corresponding to $C_{2'}$ due to the $^{2}H_{2'}$-$C_{2'}$ coupling while the incorporation of a ^{13}C in the carbonyl (C=O) of the nicotinamide moiety results in a ^{13}C-^{13}C splitting pattern for the signal corresponding to C_3 and C_4; Note the ^{31}P-^{13}C splitting pattern of $C_{5'}$ in NMN (labeled and unlabeled) absent in NR (labeled and unlabeled). **(e)** Multiple ion detection mass spectrum of ^{2}H, ^{13}C labeled NR (upper) and unlabeled NR (lower). **(f)** Multiple ion detection mass spectrum of ^{2}H, ^{13}C labeled NMN.

Supplementary Figure 8

a b c

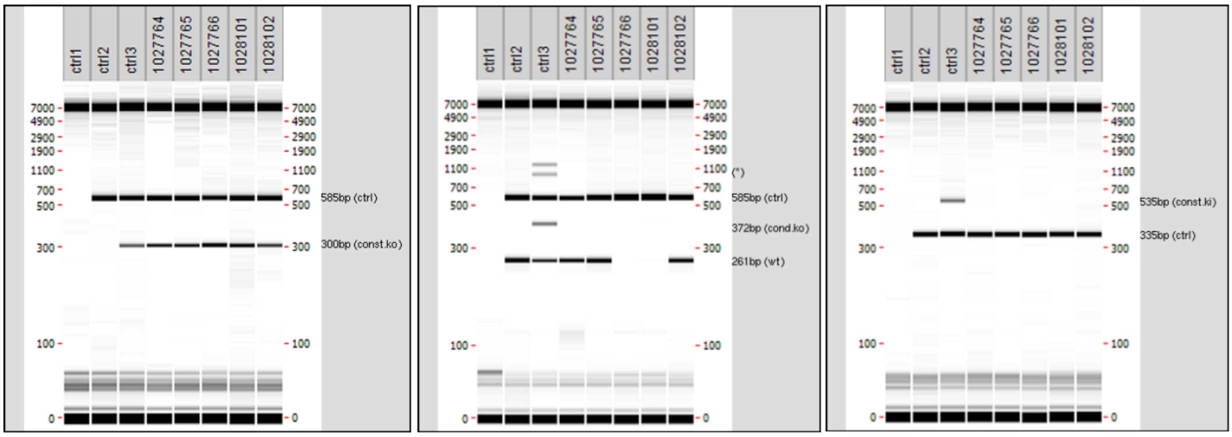

Supplementary Figure 8. NRK1KO mouse generation. Genomic DNA was extracted from tail biopsies and analyzed by PCR. The following templates were used as controls: H20 (ctrl1), wildtype genomic DNA (ctrl2), positive DNA sample (ctrl3). The amplification of the internal control fragment - 585bp (ctrl) - with oligos 1260_1 and 1260_2 confirms the presence of DNA in the PCR reactions (amplification of the CD79b wildtype allele, nt 17714036-17714620 on Chromosome 11). The amplification of the internal control fragment - 335bp (ctrl) - with oligos 1281_1 and 1281_2 confirms the presence of DNA in the PCR reactions (amplification of the CD79b wildtype allele, nt 17715045-17714730 on Chromosome 11). The PCR amplicons were analyzed using a Caliper LabChip GX device. Results are displayed as an electronic gel picture. **(a)** The fragment amplified with oligos 6075_29 and 6075_34 detects the constitutive Knock-Out (const.ko) allele. **(b)** The fragments amplified with oligos 6073_31 and 6073_32 detect the conditional Knock-Out (cond.ko) and wildtype (wt) alleles. **(c)** The fragment amplified with oligos 3253_6 and 3252_4 detects the constitutive ROSA26 Knock-In (const.ki) allele. Oligos sequences:

1260_1 GAGACTCTGGCTACTCATCC; 1260_2 CCTTCAGCAAGAGCTGGGGAC;

1281_1 GTGGCACGGAACTTCTAGTC; 1281_2 CTTGTCAAGTAGCAGGAAGA;

6075_29 AAACAATCACTGTGGCTGTGG; 6075_34 TTTCTGGATGACAGTCATTCTCC;

6073_31 ATGTGGGTTCATGGACAGC; 6073_32 GCCTCACAGCAAAAGAAGC;

3253_6 ACCAGCCAGCTATCAACTCG; 3252_4 CACCAGGTTAGCCTTTAAGCC.

Supplementary Figure 9

Full unedited gels for figure 1b

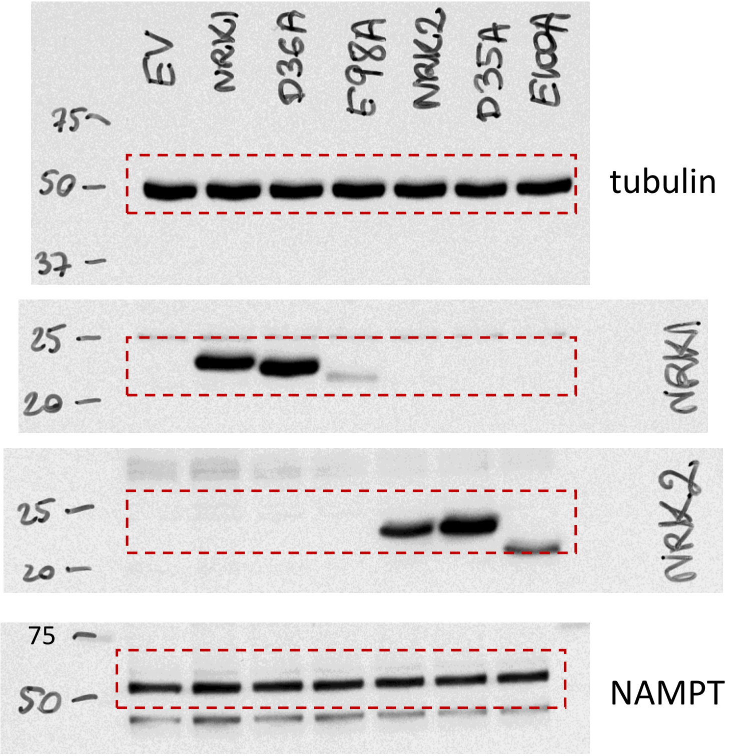

Full unedited gels for figure 2a

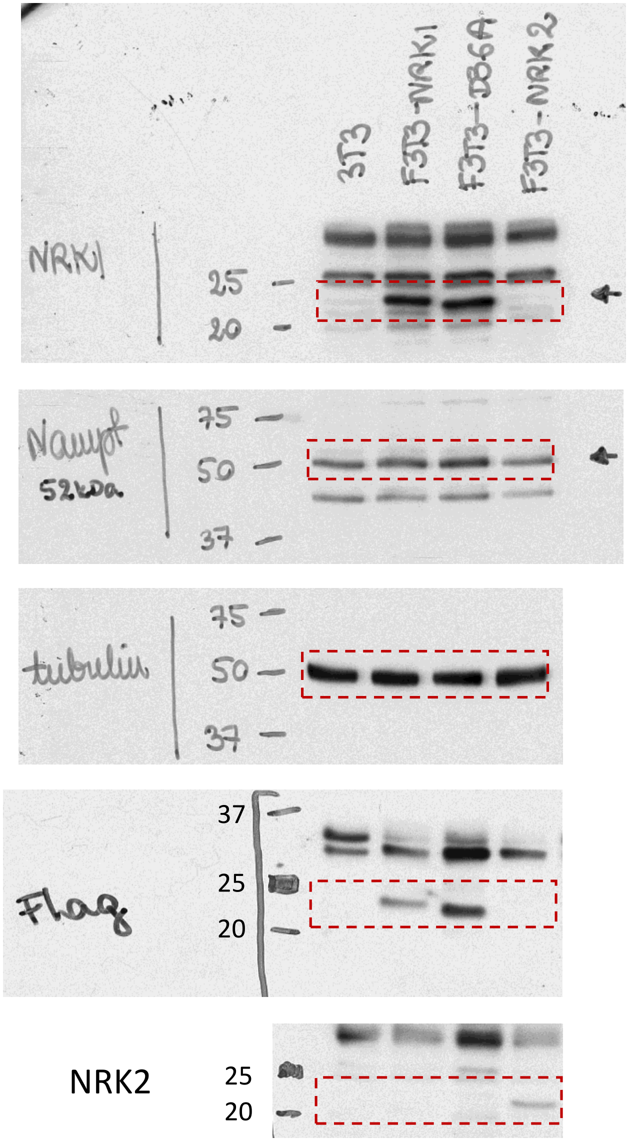

Area under the red square is represented in the final figure

Full unedited gels for figure 3a, 3c and 3d

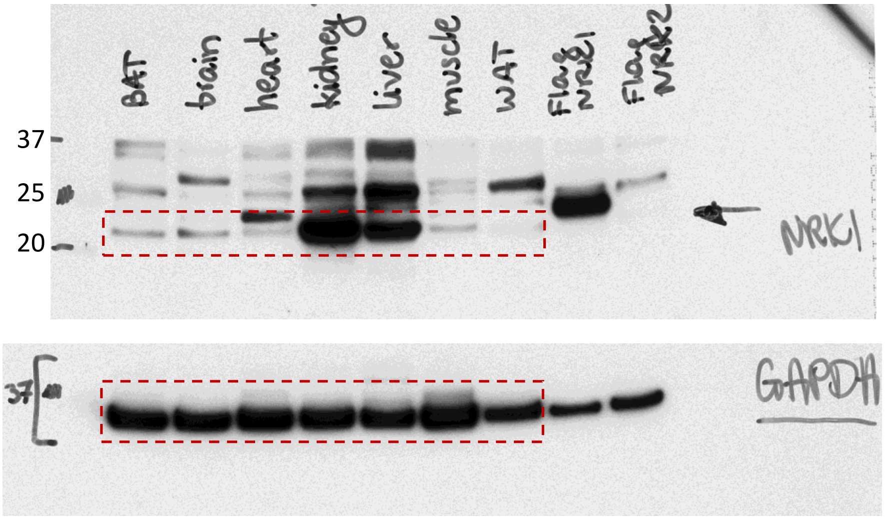

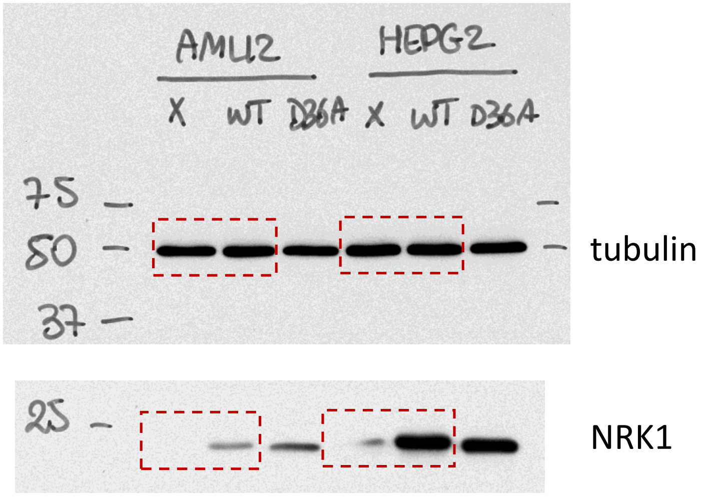

Area under the red square is represented in the final figure

Full unedited gels for figure 5a (AML12) and 5c (Hepatocytes)

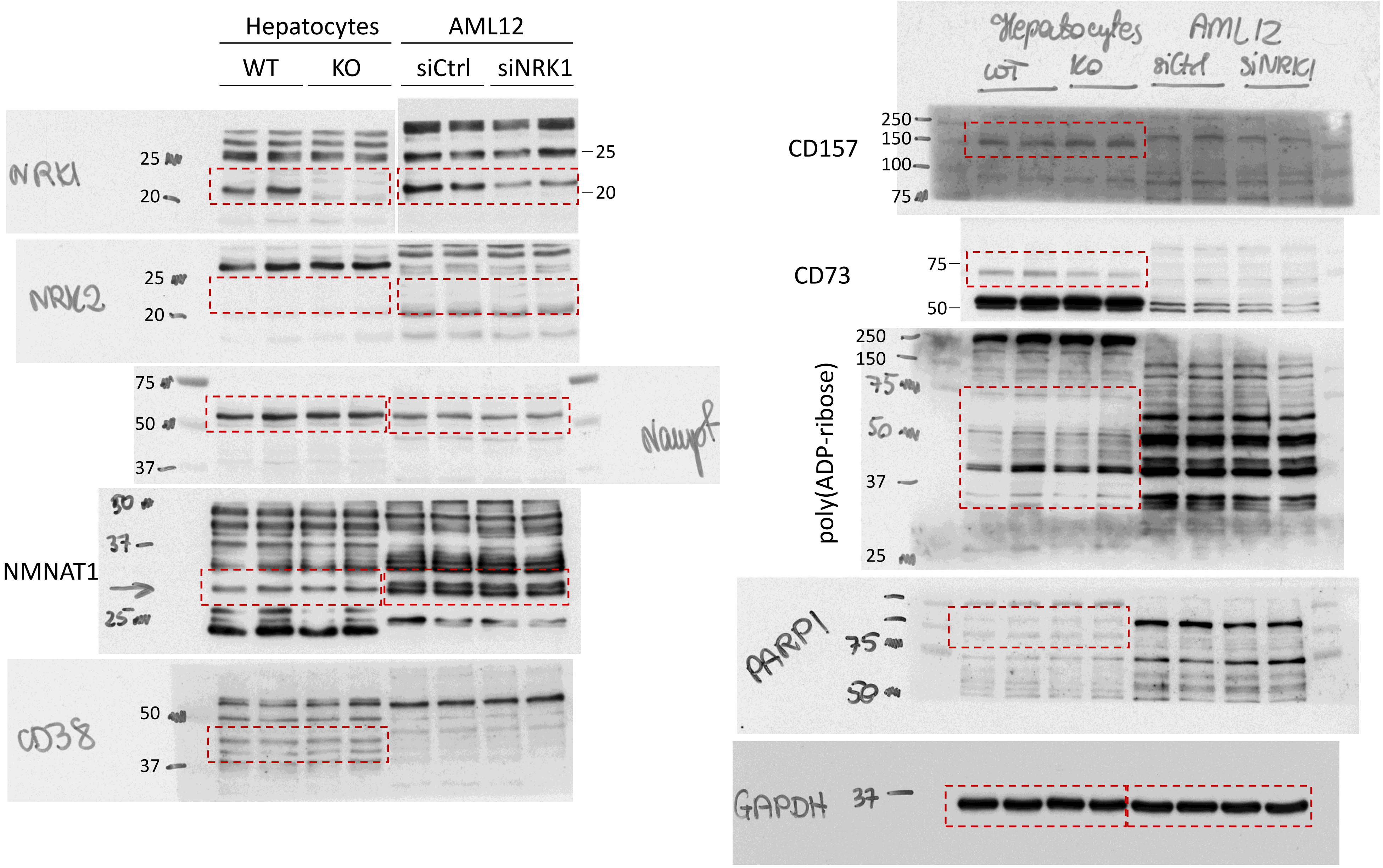

Area under the red square is represented in the final figure

Full unedited gels for Supplementary Fig. 1b

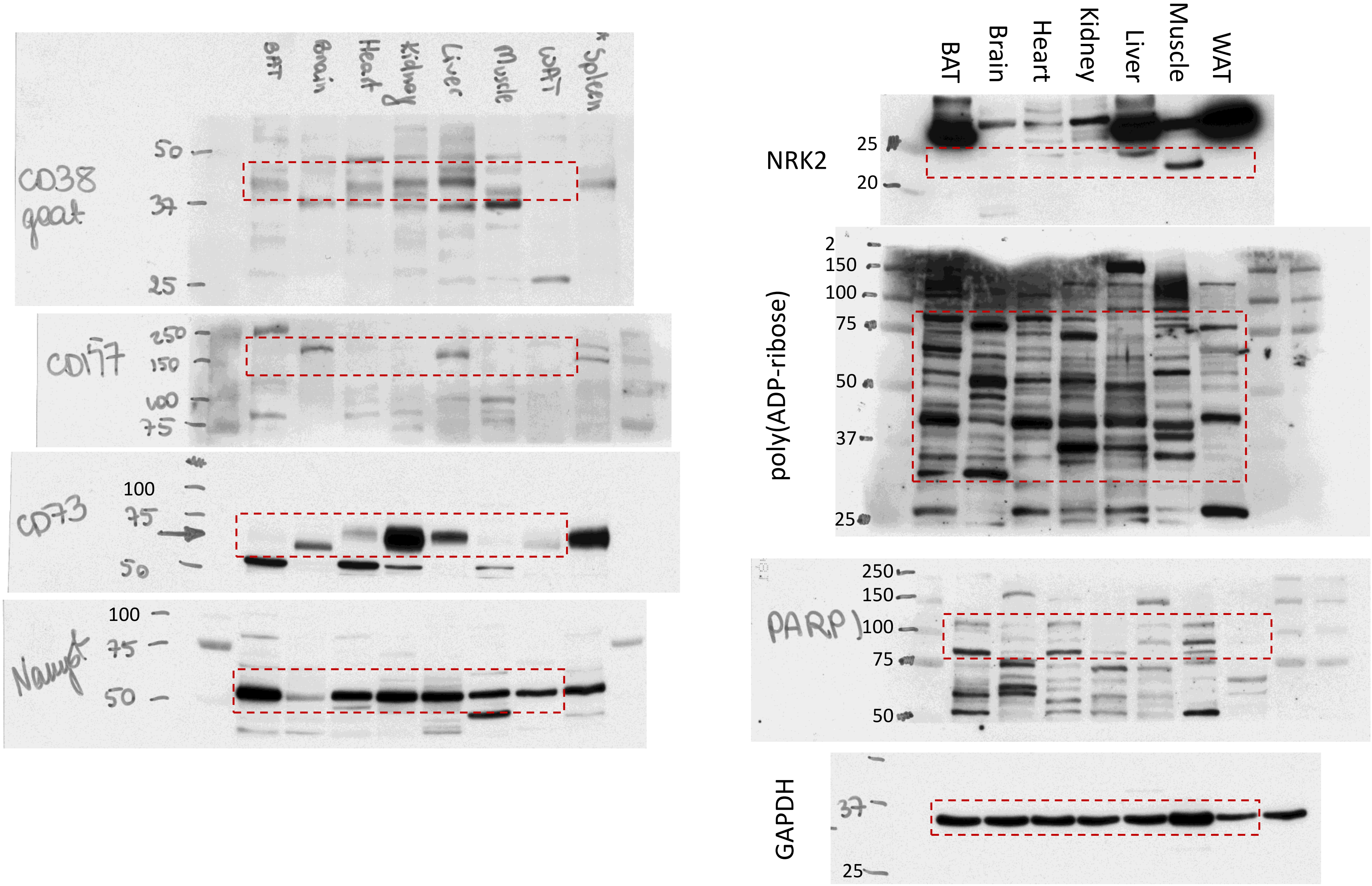

Area under the red square is represented in the final figure

Full unedited gels for Supplementary Fig. 1c and 1d

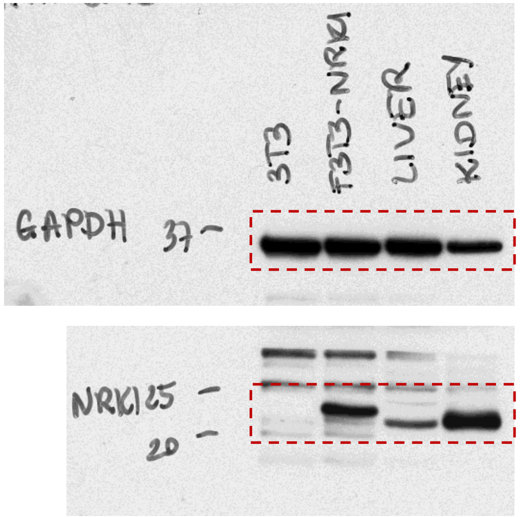

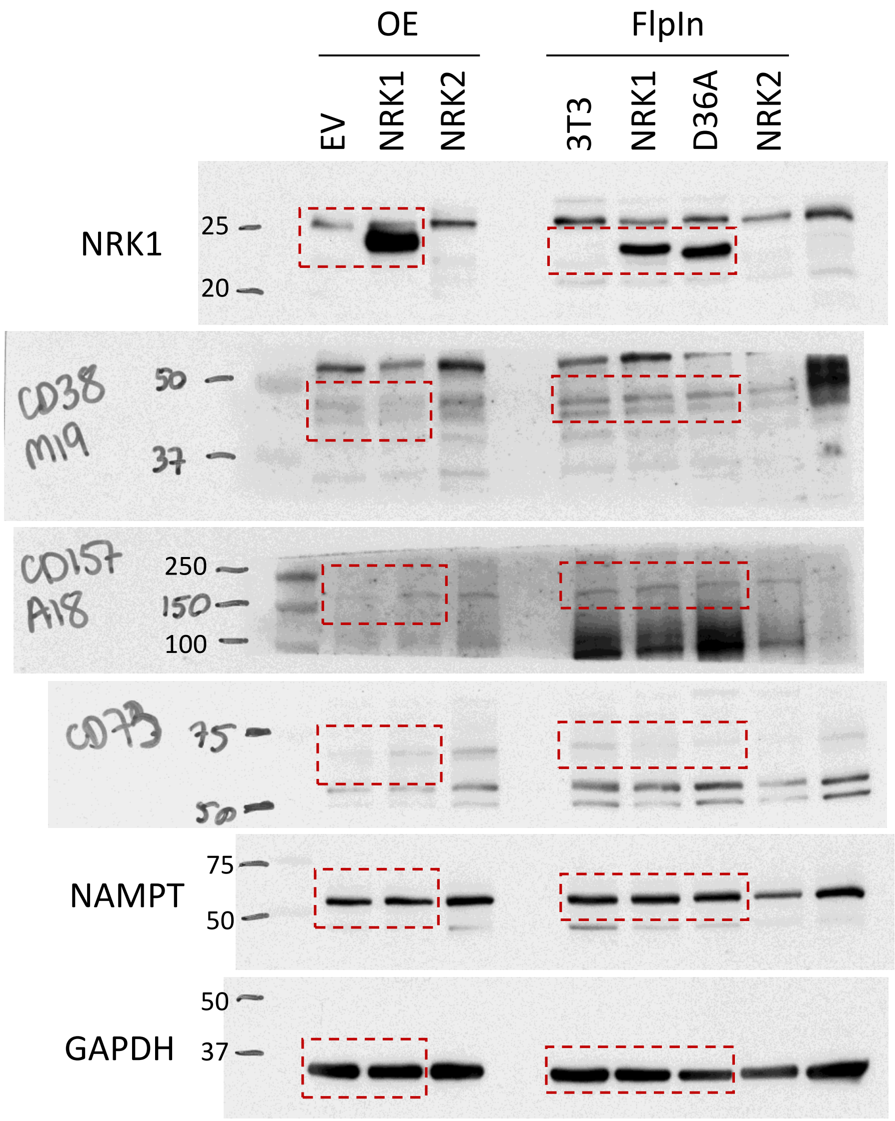

Area under the red square is represented in the final figure

Full unedited gels for Supplementary Fig. 2e

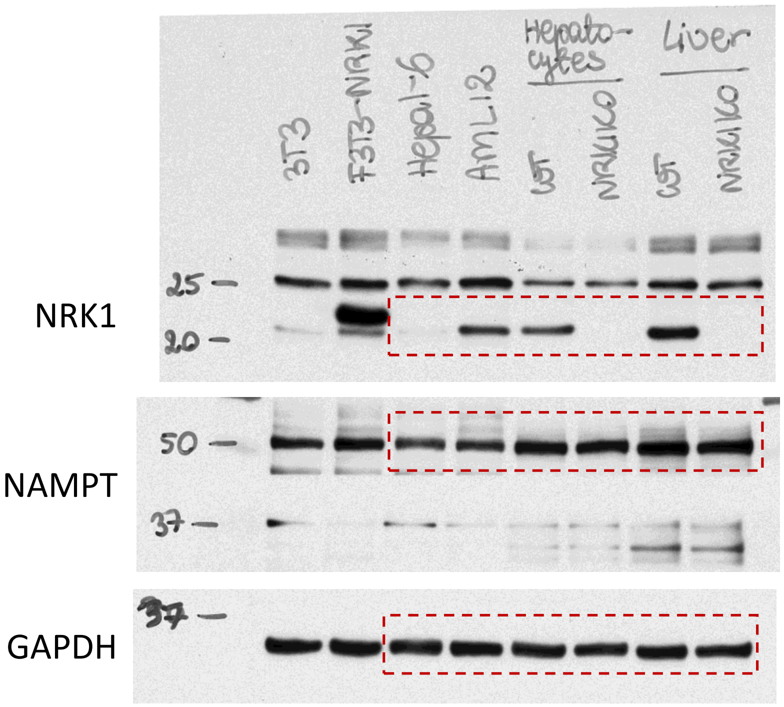

Full unedited gels for Supplementary Fig. 3d

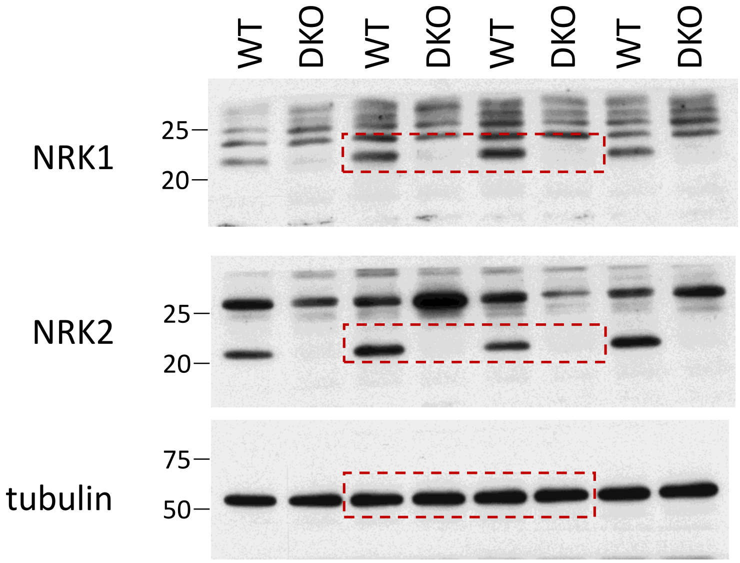

Area under the red square is represented in the final figure

Full unedited gels for Supplementary Fig. 3a

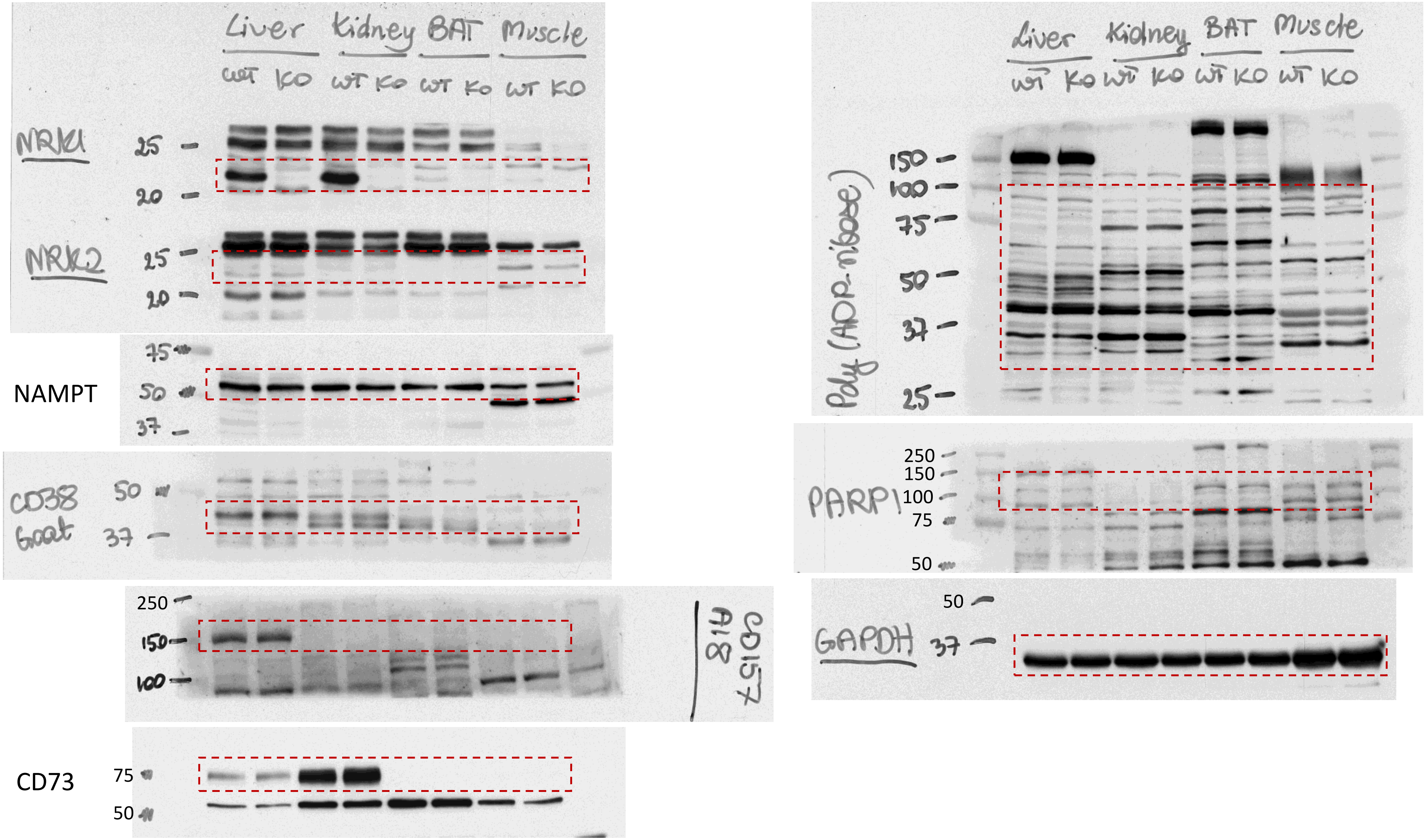

Area under the red square is represented in the final figure

Supplementary Table 1

	Liver				Plasma			
	Nam	NA	NR	NMN	Nam	NA	NR	NMN
Mean	4929749	2349	5747	628	218766	789	186	0
SD	50208	166	345	15	2399	69	25	0
CV	1.02%	7.06%	6.00%	2.46%	1.10%	8.68%	13.47%	N/A

Table 1. Standard deviation and coefficient of variation for LC-QqQ MS analysis of mouse liver and plasma samples.

Supplementary Methods

Multiple ion detection spectra of unlabeled and double labeled NR and NMN

Analyses were carried out on a Waters Acquity UPLC I-Class system (Milford, MA, USA) coupled to a Waters Xevo G2-S QTof mass spectrometer (Manchester, UK) with an electrospray ionization source operating in Resolution mode and positive mode with lock-spray interface for real time accurate mass correction. Instrument settings were as follow: source temperature was set at 120ºC, cone gas flow at 50 L.h^{-1}, desolvation temperature at 450ºC, and desolvation gas flow at 850 L.h^{-1}. The capillary voltage was set at 1.0 kV in positive mode and 1.5 kV in negative mode, respectively. Source offset was 60 (arbitrary unit). Mass spectra data were acquired in continuum mode using MSE function (low energy: 4 eV; high energy: ramp from 20 to 35 eV) over the range m/z 50-1200 with a scan time of 0.1 s. A lock-mass solution of Leucine Enkephalin (1 ng µL^{-1}) in methanol/water containing 0.1% formic acid (1:1, v/v) was continuously infused into the MS via the lock-spray at a flow rate of 10 µL min^{-1}.

The chromatographic separation was carry out on an Acquity BEH HILIC column (100 mm x 2.1 mm, 1.7 µm). The column oven temperature was set at 45ºC, injection volume at 1 µL and flow rate at 0.6 mL min-1. Mobile phase consisted of (A) water with 5 mM ammonium formate (pH 3.5) and (B) methanol with 0.025% formic acid. The gradient was set as follows: 1 min of 5% (A) followed by a linear increase from 95 to 60% (B) over 7 min, an isocratic step at 60% (B) for 0.5 min, another linear gradient to 10% (B) then returned to initial conditions 5% (A) over 0.2 min and column equilibration step at 5% (A) for 2.9 min.
